# Diagnostic Performance of ^18^F-FDG PET/CT in Papillary Thyroid Carcinoma with Negative ^131^I-WBS at first Postablation, Negative Tg and Progressively Increased TgAb Level

**DOI:** 10.1038/s41598-017-03001-7

**Published:** 2017-06-06

**Authors:** Zhong-Ling Qiu, Wei-Jun Wei, Chen-Tian Shen, Hong-Jun Song, Xin-Yun Zhang, Zhen-Kui Sun, Quan-Yong Luo

**Affiliations:** Department of Nuclear Medicine, Shanghai Jiao Tong University Affiliated Sixth People’s Hospital, Shanghai, 200233 China

## Abstract

Differentiated thyroid cancer (DTC) patients with negative serum thyroglobulin (Tg), negative ^131^I whole–body scintigraphy (^131^I-WBS) at first post-ablation and progressively increased TgAb level are a relatively rare entity in the follow-up after total thyroidectomy and radioactive iodine therapy. The value of ^18^F-FDG PET/CT in detecting the recurrence of disease in these patients has only been reported in a small case series. The goal of this study was to investigate the diagnostic accuracy of ^18^F-FDG PET/CT in detecting recurrent disease in these specific PTC patients and to identify risk factors for patients with positive ^18^F-FDG PET/CT results. Eighty-two PTC patients who had ^18^F-FDG PET/CT scans with negative Tg, negative ^131^I-WBS at first post-ablation and progressively increased TgAb levels were included. We found that the sensitivity, specificity, positive predictive value, negative predictive value, and accuracy of ^18^F-FDG PET/CT in this patient group were determined as 84%, 72%, 92%, 57% and 82%, respectively. ^18^F-FDG PET/CT scan had a good diagnostic performance and should be performed routinely in PTC patients with negative Tg, negative ^131^I-WBS at first postablation and progressively increased TgAb level, especially when span for progressively increased TgAb level ≥ 3 years and/or progressively increased TgAb value up to 150 IU/mL.

## Introduction

Differentiated thyroid cancer (DTC) is becoming more and more common in the United States between 1975 and 2012, with an estimated 62,450 new cases in 2015^1^. Despite the high overall survival rate and good prognosis, the recurrence rate of DTC is not negligible and ranges from 14% to 23%^2, 3^. Therefore, detection of persistent or recurrent disease is very important in DTC management and follow-up. ^131^I whole–body scintigraphy (^131^I-WBS), measurement of serum thyroglobulin (Tg) level and neck ultrasonography are mainstream approaches for detecting persistent or recurrent disease after total or near-total thyroidectomy and radioiodine remnant ablation^4^. Serum Tg level is the most sensitive and reliable marker indicating persistent or recurrent disease in the follow-up of DTC because serum Tg only originated from differentiated thyroid cancer cells^5^.

According to the current American Thyroid Association (ATA) guideline, negative serum Tg, defined as the low serum Tg levels during TSH suppression (Tg < 0.2 ng/mL) or after stimulation (Tg < 1 ng/mL) after total or near-total thyroidectomy and radioiodine remnant ablation, suggests the disease-free status for DTC patients in the follow-up^6^. However, antithyroglobulin antibody (TgAb) can interfere with the measurement of Tg and reduce the accuracy of Tg as a predictor of DTC activity. In a previous study, TgAb was present in 10–25% of patients with PTC^7, 8^. Therefore, negative Tg with the presence of positive TgAb could lead to a clinical dilemma in terms of therapeutic decision and follow-up.

Whether or not elevated serum TgAb concentrations can be used as a surrogate marker of persistent or recurrent disease remains controversial^9, 10^. Some argued that TgAb levels did not predict disease status in DTC because TgAb production primarily arises in coexisting lymphocytic thyroiditis or Graves’ disease in DTC patients^11^. It has been reported that only the progressively increased TgAb level was useful for predicting clinical recurrence or persistence of Tg-negative patients with PTC^12, 13^.

^18^F-fluorodeoxy-D-glucose positron emission tomography/computed tomography (^18^F-FDG PET/CT) is routinely performed to search for the recurrent or persistent disease in patients with DTC^14^. However, only a few studies including a small case series evaluated the value of ^18^F-FDG PET/CT in DTC patients who have negative ^131^I-WBS, negative Serum Tg, and increased TgAb titer^15, 16^.

In the present study, we aimed to investigate the diagnostic accuracy of ^18^F-FDG PET/CT, performed over one year after their first remnant ablation, in detecting recurrent disease of PTC in a relatively large clinical samples of patients with negative Tg, negative ^131^I-WBS at first postablation and progressively increased TgAb level. Moreover, we also identified the correlation of clinical and pathological factors with positive ^18^F-FDG PET/CT findings in this specific cohort.

## Results

### Patient’s characteristics

According to the inclusion and exclusion criteria, eighty-two PTC patients who underwent ^18^F-FDG PET/CT scans with negative Tg, negative ^131^I-WBS at first post-ablation and progressively increased TgAb level were confirmed and included. Of them, 58 (71%) PTC patients with serum Tg levels < 0.2 ng/mL (TSH suppression) and 24 (29%) PTC patients with serum Tg levels < 1 ng/mL (TSH stimulation >30 IU/mL) at 6 months after first remnant ablation. Serum Tg levels were always <0.2 ng/mL under TSH suppression for all these patients in the follow-up. The characteristics of the study cohort at diagnosis of recurrent PTC were shown in Table 1.

**Table 1:** Patients’ characteristics.

N = 82 patients	
**Sex**	
Male	32 (39%)
Female	50 (61%)
**Age** (**years**)	
≥45	35 (43%)
<45	47 (57%)
**Mean age** (**range**)	48 (17–76)
**Subtypes of PTC**	
Classical	71 (86%)
Follicular variant	4 (5%)
Aggressive	7 (9%)
**Tumor size** (**mm**)	
≤20	21 (26%)
20–40	43 (52%)
>40	18 (22%)
**Extrathyroid extension**	19 (23%)
**Central lymph node dissection**	
None	5 (6%)
Central only	26 (32%)
Central + ipsilateral only	35 (43%)
Central + bilateral	16 (19%)
**Bilateral tumor**	21 (26%)
**Multifocal tumor**	25 (30%)
**N stage**	
N0	9 (11%)
N1a	39 (47%)
N1b	27 (33%)
Nx	7 (9%)
**Pathology with lymphocytic thyroiditis**	13 (16%)
**Mean TgAb level prior to elevation**, **IU/mL** (**range**)	102 (12–1902)
**Mean TgAb level at diagnosis**, **IU/mL** (**range**)	479 (98–3726)

### ^18^F-FDG PET/CT finding

**Figure 1:**
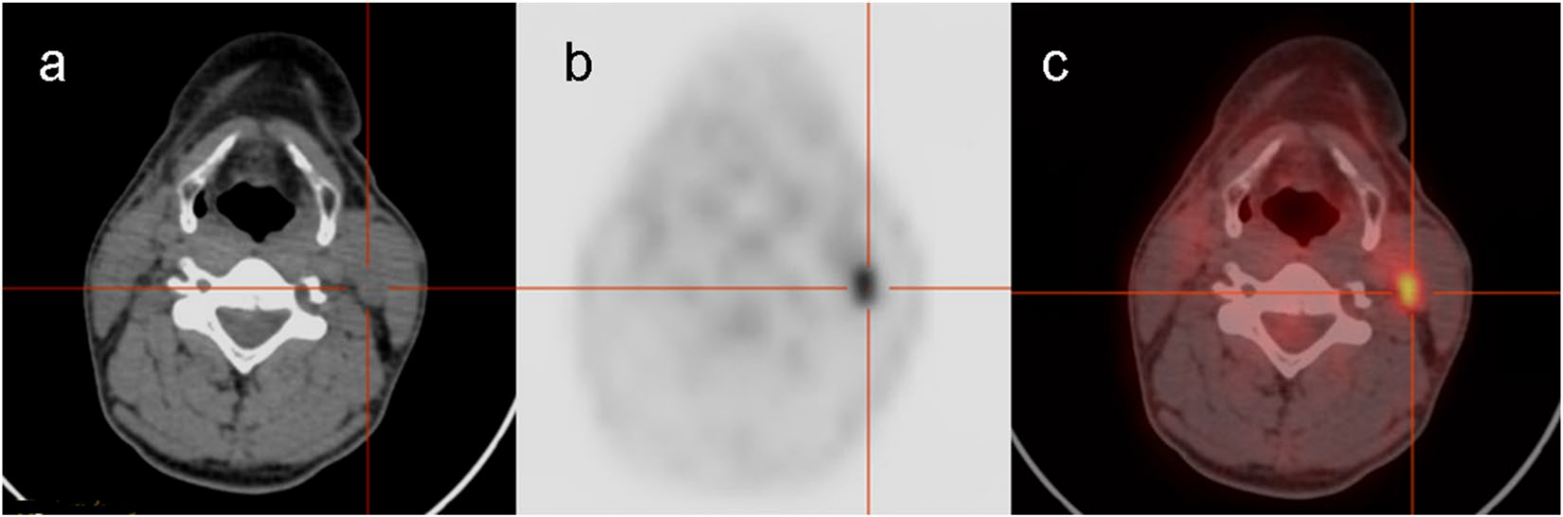
A true-positive lesion on the left neck region was detected by ^18^F-FDG PET/CT. A 35-year old woman underwent total thyroidectomy with central lymph node dissection on the right neck region and radioiodine ablation for remnant PTC and thyroid tissue. ^131^I-WBS obtained 5 days after an oral administration of 3.7 GBq of ^131^I showed negative finding. Six months after ablation, the patient had negative Tg (<0.1 ng/mL) but abnormal TgAb of 108 IU/mL at TSH suppression status. Subsequently, during the follow-up 3.5 years later, TgAb level progressively increased from 108 IU/mL to 623 IU/mL. ^18^F-FDG PET/CT revealed ^18^F-FDG-avid nodal lesion with SUV_max_ of 4.7 in the left neck (**a**,**b** and **c**, crossing line). Surgical pathology confirmed the metastatic nodal lesion from PTC after left neck dissection. The patient had markedly decreased TgAb level afterwards.

**Figure 2:**
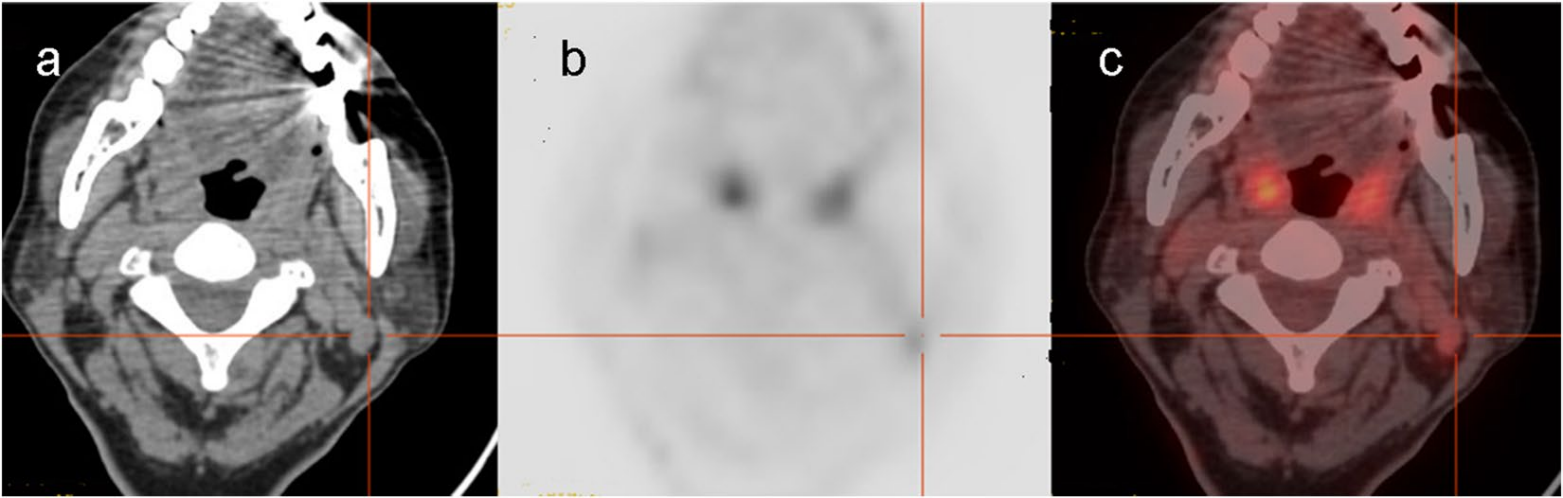
A false-positive lesion on the left neck region was revealed by ^18^F-FDG PET/CT. A 62-year-old woman underwent total thyroidectomy with radical left neck dissection for PTC followed by radioiodine therapy with 3.7 GBq of ^131^I. Three days later, post-therapy ^131^I WBS was performed and showed negative results. Six months after ablation. The serum Tg level was 0.18 ng/mL and TgAb level was 46 IU/mL at TSH suppression status. TgAb level was stable for 4.2 yr after ^131^I therapy. But subsequently, TgAb gradually increased at TSH suppression status. TgAb level elevated from 51 IU/mL to 137 IU/mL in the next follow-up of 2.2 years. ^18^F-FDG PET/CT demonstrated increased foci radiotracer uptake in the left submandibular region (**a**,**b**, crossing line), which was localized by CT image to the left submandibular lymph nodes with SUV_max_ of 2.6 (**c**, crossing line). However, infectious lymph node was diagnosed on histopathology after surgery.

Of 82 patients with ^18^F-FDG PET/CT findings, 59 (72%) patients had results interpreted as positive and 23 (28%) patients as negative. In 59 cases with positive ^18^F-FDG PET/CT findings, 54 (91.5%) patients were classified as true-positive confirmed pathologically by surgical specimens (Fig. 1). Neck US was performed in all patients before ^18^F-FDG PET/CT scan. 39 patients with 51 lymph node metastases were found in the neck, among which 34 patients with 42 lesions were detected by neck US. Two cases with 2 lymph nodes metastases, one case with 1 lymph node metastasis and 6 cases with 7 lymph node metastases were found in pharyngeal space, parotid, and mediastinum, respectively, all of which were detected by contrast-enhanced CT after ^18^F-FDG PET/CT scanning. 4 patients with ^18^F-FDG PET lung metastases were diagnosed, all of which were detected and confirmed by chest CT (Table 2). 7 false-positive lesions were found on ^18^F-FDG PET/CT scan in 5 patients in the neck, 5 FDG-avid lesions in 4 patients and 2 FDG-avid lesions in 1 patient were diagnosed as reactive infection of lymph node and hyperplasia of lymph nodes (Fig. 2). All these patients had suspicious lymph nodes for recurrence on the neck US.

**Table 2:** Sites for recurrent diseases in 54 patients with true-positive ^18^F-FDG PET/CT findings.

Sites of recurrent diseases	No. of patients/foci
**Cervical lymph nodes**	**39/51**
Thyroid bed	5/5
Left	16/22
Right	18/24
**Parapharyngeal lymph nodes**	**2/2**
**Parotid lymph nodes**	**1/1**
**Mediastinal lymph nodes**	**6/7**
**Lungs**	**4**

In 23 cases with negative ^18^F-FDG PET/CT findings, 10 patients were interpreted as false-negative, among which 13 lymph node metastases from 8 patients were detected in the neck and 2 lymph node metastases from the remaining 2 patients were found in the mediastinum. All the 10 patients with false-negative results received ^131^I treatment once again. 2 cases with 2 neck lymph node metastases and one case with 1 mediastinal lymph node metastasis were diagnosed by ^131^I-WBS combined with ^131^I-SPECT/CT after ^131^I treatment. The remaining 7 patients with 10 lesions who had negative ^131^I-WBS results were confirmed by surgical pathology. All these patients had suspicious lymph node recurrence on the neck US or contrast-enhanced CT of chest (Table 3).

**Table 3:** Clinical and pathological characteristics for 10 patients with false-negative ^18^F-FDG PET/CT findings.

Patient No.	Age/Sex	Histology	Serum TgAb level prior to elevation (IU/mL)	Serum TgAb level at diagnosis (IU/mL)	Sites of recurrent diseasSe (No.)	Neck US	Contrast-enhanced CT of Chest	Progressive increase in TgAb level (years)	Confirmation methods
1	27/Male	Classical	76	148	Left neck (1)	Suspicious	—	2.2	Histopathology
2	47/Female	Classical	29	134	Left neck (1)	Suspicious	—	3.6	Histopathology
3	51/Female	Classical	73	126	right neck (1)	Suspicious	—	3.0	^131^I-WBS + SPECT/CT
4	57/Female	Aggressive	98	142	right neck (2)	Suspicious	—	5.5	Histopathology
5	32/Male	Classical	12	98	Meditational (1)	—	Suspicious	4.2	Histopathology
6	21/Female	Classical	45	122	right neck (1)	Suspicious	—	4.4	^131^I-WBS + SPECT/CT
7	36/Female	Classical	89	132	right neck (2)	Suspicious	—	2.8	Histopathology
8	42/Female	Classical	58	179	right neck (1)	Suspicious	—	3.3	Histopathology
9	69/Male	Classical	324	419	Left neck (4)	Suspicious	—	4.6	Histopathology
10	41/Female	Classical	171	635	Meditational (1)	Suspicious	Suspicious	5.6	^131^I-WBS + SPECT/CT

Of 13 true-negative patients, neck US finding was suspicious for recurrence in 7 patients with 12 lesions, but disease recurrence was not detected by surgical excision. The remaining 6 patients underwent ^131^I treatment once again and negative findings were shown on the ^131^I-WBS, and recurrent diseases were not be detected by US neck and chest contrast-enhanced CT in these 6 patients. In addition, bone metastases from PTC weren’t detected on the ^99m^Tc-bone scan, but TgAb levels were gradually rising in the follow-up. These 6 patients were also classified as true-negative.

### Factors influencing positive ^18^F-FDG PET/CT results

Patient age, sex, subtypes of PTC, tumor size, extrathyroid extension, bilateral tumor, multifocal tumor, whether patient with neck lateral dissection at initial surgery, N stage, whether pathology with lymphocytic thyroiditis not significantly associated with positive ^18^F-FDG PET/CT results (P > 0.05). TgAb level at diagnosis and span for progressively increased TgAb level were statistically significant in predicting positive ^18^F-FDG PET/CT findings (P < 0.05). Compared with TgAb level <150 IU/mL at diagnosis and span for progressively increased TgAb level less than 3 years, univariate regression analysis showed that OR value of TgAb level ≥ 150 IU/mL at diagnosis and span for progressively increased TgAb level longer than 3 years were as much as 4.18 [CI:1.52–11.54] and 3.60 [CI:1.24–10.41] times for progressively increased TgAb level (Table 4).

**Table 4:** Risk factors for positive ^18^F-FDG PET/CT results in this specific cohort.

Factors	Positive FDG PET/CT (Yes/Total)	Univariate analysis OR (CI 95%)	*χ* ^2^	p
**Sex**			**0**	**1**
Male	23/32 (72%)	1		
Female	36/50 (72%)	1.00[0.37–2.67]		
**Age**(**years**)			**0**.**17**	**0**.**69**
≥45	26/35 (74%)	1		
<45	33/47 (70%)	1.22[0.46–3.27]		
**Subtypes of PTC**			**0**.**09**	**0**.**77**
Classical	52/71 (64%)	1		
Others	7/11 (73%)	1.56[0.41–5.95]		
**Tumor size**(**mm**)			**3**.**36**	**0**.**19**
≤20	12/21 (57%)	1		
20–40	34/43 (79%)	0.83[0.91–8.81]		
>40	13/18 (72%)	1.95[0.51–7.49]		
**Extrathyroid extension**			**0**.**15**	**0**.**70**
Yes	13/19 (68%)	1		
No	46//63 (73%)	1.25[0.41–3.81]		
**Bilateral tumor**			**0**.**004**	**0**.**951**
Yes	15/21 (71%)	1		
No	44/61 (72%)	1.04[0.36–3.11]		
**Multifocal tumor**			**1**.**13**	**0**.**29**
Yes	16/25 (64%)	1		
No	43/57 (75%)	1.73[0.63–4.77]		
**Neck lateral dissection**			**0**.**74**	**0**.**39**
Yes	35/51 (69%)	1		
No	24/31 (77%)	1.57[0.56–4.39]		
**N stage**			**2**.**43**	**0**.**12**
N0-Nx	9/16 (56%)	1		
N1	50/66 (76%)	2.43[0.78–7.58]		
**Pathology with lymphocytic thyroiditis**			**0**.**83**	**0**.**36**
No	51/69 (74%)	1		
Yes	8/13 (62%)	0.57[0.16–1.95]		
**TgAb level at diagnosis** (**IU/mL**)			**8**.**13**	**<0**.**001**
<150	16/30 (53%)	1		
≥150	43/52 (83%)	4.18[1.52–11.54]		
**Progressive increase in TgAb level** (**years**)			**5**.**9**	**0**.**02**
<3	26/43 (61%)	1		
≥3	33/39 (85%)	3.60[1.24–10.41]		

### Diagnostic accuracy of ^18^F-FDG PET/CT scans

In all these patients, the true-positive, false-positive and false-negative, true-negative cases of ^18^F-FDG PET/CT findings were 54, 5, 10, and 13, respectively. The sensitivity, specificity, positive predictive value, negative predictive value, and accuracy of ^18^F-FDG PET/CT in this patient group were determined as 84%, 72%, 92%, 57% and 82%, respectively (Table 5).

**Table 5:** ^18^F-FDG PET-CT findings of the included patients.

^18^F-FDG PET-CT	All patients	TgAb level at diagnosis (IU/mL)		Progressive increase in TgAb level (years)	
	82	<150	≥150	<3	≥3
True-positive	54	13	41	22	32
False-positive	5	3	2	4	1
False- negative	10	8	2	7	3
True-negative	13	6	7	10	3
Sensitivity	84%	62%	95%	76%	91%
Specificity	72%	67%	78%	71%	75%
Positive predictive value	92%	81%	95%	85%	97%
Negative predictive value	57%	43%	78%	59%	50%
Accuracy	82%	63%	92%	74%	90%

When comparing different TgAb levels at diagnosis, the sensitivity, specificity, positive predictive value, negative predictive value, and accuracy of ^18^F-FDG PET/CT for patients whose TgAb levels ≥ 150 IU/mL at diagnosis is higher than for those whose TgAb levels < 150 IU/mL at diagnosis. The sensitivity, specificity, positive predictive value, negative predictive value, and accuracy increased from 62% to 95%, from 67% to 78%, from 81% to 95%, from 43% to 78% and from 63% to 94% respectively (Table 5). When comparing different span for progressively increased TgAb level, the sensitivity, specificity, positive predictive value, and accuracy of ^18^F-FDG PET/CT for span for progressively increased TgAb level longer than 3 years at diagnosis were superior to that less than 3 years at diagnosis, the sensitivity, specificity, positive predictive value, and accuracy of ^18^F-FDG PET/CT scan increased from 76% to 91%, from 71% to 75%, from 85% to 97%, from 74% to 90%. However, the negative predictive value of ^18^F-FDG PET/CT for patients whose span for progressively increased TgAb level ≥ 3 years at diagnosis was inferior to that <3 years at diagnosis. The negative predictive value decreases from 59% to 50% (Table 5).

## Discussion

Our study demonstrated that ^18^F-FDG PET/CT was a useful method for detecting recurrent disease in PTC patients with negative Tg, negative ^131^I-WBS at first post ablation and progressively increased TgAb level. In the current study, the sensitivity, specificity, positive predictive value, negative predictive value, and accuracy of ^18^F-FDG PET/CT for this patient group were confirmed as 84%, 72%, 92%, 57% and 82%, respectively. The diagnostic performance of ^18^F-FDG PET/CT scanning for detecting the recurrent thyroid cancer with negative Tg, negative ^131^I-WBS and increased TgAb has been reported several retrospective studies^16–21^ (Table 6). The sensitivity, specificity, positive predictive value, negative predictive value, and accuracy of ^18^F-FDG PET/CT examination for these patients ranged from 75% to 100%, from 50% to 100%, from 50% to 100%, from 50% to 100%, from 25% to 100% and from 72.7% to 88.4%. The difference within these several studied may reflect the heterogeneity of the number and patients included, definition of Tg negativity, selection criteria for TgAb level, follow-up time, specific ^18^F-FDG PET/CT technique used, or the reference standard against which the accuracy of ^18^F-FDG PET/CT scan were analyzed. Among them, five articles have a small number of cases (16, 18–21), so the results were very easy to produce deviation. The largest ^18^F-FDG PET/CT series to date was the retrospective study by Asa *et al*. and included 40 DTC patients, the sensitivity, specificity, positive predictive value, negative predictive value, and accuracy in this large study were 78.5%, 50%, 91.6%, 25% and 75%^21^, all of which lower than those reported by ours. This difference may reflect possibly definition of Tg negativity, selection criteria bias of TgAb and follow-up time. In our study, Negative serum Tg was defined as Tg < 0.2 ng/mL (TSH suppression) or Tg < 1 ng/mL (after stimulation) at 6 months after the first ^131^I remnant ablation, while Asa *et al*. considered that the PTC patients had negative serum Tg level as Tg ≤ 1 ng/mL (TSH suppression) or Tg ≤ 2 ng/mL (after stimulation) in the follow-up period after total-near total thyroidectomy and ^131^I ablation^21^. If DTC patients with coexistent clinical Hashimoto thyroiditis Graves’ disease, or focal autoimmune thyroiditis, all TgAb disappeared more slowly and the median disappearance time was 3 years for TgAb after total thyroidectomy and radioiodine ablation^22^. Therefore, increased TgAb level without upward trend in a short follow-up time might not be viewed as persistent or recurrent diseases of DTC. In our cases, increased TgAb level without upward trend has been excluded. Although Asa *et al*. selected the TgAb standard for persistently/progressive increased TgAb, whether the content of the article including increased TgAb level without upward trend was unclear^21^. Otherwise, it was reported that the TgAb levels measured 6–12 months after ablation therapy were significantly rising in the DTC patients with residual disease compared to those with no residual disease^23^. Whether this situation for DTC patients ruled out was indeterminate for the study by Asa *et al*. because DTC patients in their groups have relatively short follow-up times (9–36 months).

**Table 6:** Diagnostic efficacies of ^18^F-FDG PET/CT in patients with DTC and elevated serum TgAb in other studies.

Author	Publication year	No. of patients	Sensitivity	Specificity	Positive predictive value	Negative predictive value	Accuracy
Chung *et al*.^17^	2002	26	84.6%	92.3%	—	—	88.4%
Viedma *et al*.^18^	2011	22	100%	62.5%	50%	100%	72.7%
Bogsrud *et al*.^19^	2011	15	83.3%	100%	100%	71.4%	70.6%
Ozkan *et al*.^20^	2012	31	75%	76%	75%	86%	80%
Ozkan *et al*.^16^	2013	10	100%	50%	75%	100%	80%
Asa *et al*.^21^	2014	40	78.5%	50%	91.6%	25%	75%

In our study, of 59 cases with positive ^18^F-FDG PET/CT finding, 54 (91.5%) patients were classified as true-positive confirmed pathologically by surgical resection and 5 patients were diagnosed for false-positive on ^18^F-FDG PET/CT scans. False-positive ^18^F-FDG uptake in the neck was often caused by several sources including muscle, brown fat, salivary glands, vocal cords, tonsils, and other lymphoid tissues. Moreover, reactive hyperplasia lesions, inflammatory lesions and benign tumors can also lead to FDG uptake^24^. All false-positive uptakes were located in the neck, which was similar to what Ozkan *et al*. reported^16^. Ozkan *et al*. considered that it was difficult to distinguish recurrent lesions from false-positive lesions using SUV_max_ ≥ 2.5 t in the neck region because of overlapping SUVs between them^16^. Therefore, the criterion for positive lesion was accepted as ^18^F-FDG uptake greater than that of the normal surrounding tissue or when the SUV_max_ was ≥ 2.5 in our study.

With respect to distant metastases from included PTC patients, only 4 cases with lung metastases were detected by ^18^F-FDG PET/CT scan, all of which showed that PTC patients with negative Tg, negative ^131^I-WBS and progressively increased TgAb level weren’t prone to distant metastases. It was very possible because distant metastases of DTC produced more serum Tg which couldn’t be completely interfere by TgAb^25^. All of them received additional ^131^I therapy and showed negative post therapy ^131^I-WBS scan results.

Of 23 cases with negative ^18^F-FDG PET/CT finding, 10 patients were interpreted as false-negative. Suspicious recurrent lymph node metastases were detected on the neck, which may suggest that negative ^18^F-FDG PET/CT finding may represent a small or well-differentiated metastatic lesions^26^. Subsequently, these patients underwent empirical ^131^I therapy using 150 mCi. After 3–5 days, 3 patients with metastatic lesions were detected and confirmed by ^131^I-WBS combined with ^131^I-SPECT/CT. The remaining 7 patients showed negative ^131^I-WBS and recurrent lesions were confirmed pathologically by surgical resection. In the current study, there are also 13 true-negative patients with progressively increased TgAb level, while 7 out of these patients were surgically confirmed as disease free, and the remaining 6 cases were confirmed by follow-up.

With regard to true-negative and false-positive finding in these patients, it was not uncertain whether a few other reasons made serum TgAb rise continuously except for the recurrent diseases of PTC, for that a few other diseases could lead to the increased TgAb, such as type 1 diabetes, rheumatoid arthritis, pernicious anemia, collagen vascular diseases, scleroderma, chronic urticarial, autoimmune hyperthyroidism and increasing age in healthy women has been reported^22, 27–30^.

In our series, univariate analysis revealed that different TgAb level at diagnosis and span for progressively increased TgAb level was related to the positive ^18^F-FDG PET/CT finding. The results showed that the sensitivity, specificity of ^18^F-FDG PET/CT when TgAb level ≥ 150 IU/mL at diagnosis and span for progressively increased TgAb level ≥ 3 years were clearly higher than that when TgAb level < 150 IU/mL at diagnosis and span for progressively increased TgAb level 3 years, respectively. In previous studies, a varying prevalence of TgAb value for predicting persistent or recurrent of DTC after total thyroidectomy has been reported. Chung *et al*. considered a serum TgAb level below 100 U/mL as negative, and found elevated TgAb levels in 22.6% of DTC patients after ^131^I ablation^17^. Seo *et al*. reported that recurrence for DTC was more frequent in patients who showed a persistently elevated TgAb level over 140 U/mL^31^. While other studied have defined TgAb level of 6–100 U/mL as positive^32, 33^. In our study, different TgAb levels were used as cutoff values to evaluate the diagnostic performances of ^18^F-FDG PET/CT scan in the detection of recurrent PTC. ^18^F-FDG PET/CT results could be affected by lesion size, false-positive finding and degree of differentiation of PTC and so on, therefore, above research results for TgAb level may be not related to the positive ^18^F-FDG PET/CT finding. Our study also showed that longer span for progressively increased TgAb level (≥3 years) rather than shorter pan for progressively increased TgAb level (<3 years) indicated a higher sensitivity, specificity of ^18^F-FDG PET/CT scanning in detecting recurrent PTC, suggesting the recurrent diseases of these PTC patients developed more slowly and have a relatively good prognosis.

However, several limitations of this study should be discussed. First, of 13 true negative patients, 6 patients weren’t confirmed having metastases by pathology. These cases were classified as true negative through follow-up. In the course of follow-up, these 6 patients showed negative findings on the post-therapy ^131^I-WBS, neck US and contrast-enhanced chest CT, ^99m^Tc-MDP bone scan, but some occult lesions may still not be found. Second, Of 10 false-negative patients, 3 patients were confirmed by ^131^I-WBS combined with ^131^I-SPCET/CT rather than the results of pathology, because ^131^I-SPECT/CT effectively excluded residual noncancerous thyroid tissue located outside the thyroid bed (substernal goiter or ectopic foci along the thyroglossal duct), physiologic uptake in non-thyroidal tissues, and contamination^34, 35^. The retrospective nature of the data in the present study may be another limitation.

## Conclusions

Our study demonstrated that the ^18^F-FDG PET/CT scanning had a good diagnostic performance in the selected PTC patients with negative Tg, negative ^131^I-WBS at first postablation and progressively increased TgAb level. Span for progressively increasing TgAb level and TgAb level at diagnosis were closely associated with positive ^18^F-FDG PET/CT findings. Therefore, ^18^F-FDG PET/CT scanning could be performed routinely for PTC patients with negative Tg, negative ^131^I-WBS at first postablation ablation and progressively increased TgAb level, especially for those whose span for progressively increased TgAb level ≥3 years and/or progressively increased TgAb value up to 150 IU/mL.

## Patients and Methods

### Patients

This retrospective study was approved by our institutional review board. Informed consents have been waived for most patients except for two patients, whose SPECT/CT images were used in the current study. All methods were performed in accordance with the relevant guidelines and regulations. Files of consecutive 7843 patients treated with ^131^I between January 2005 and January 2014 were reviewed. Clinical follow-up data of 1257 patients with DTC who underwent ^18^F-FDG PET/CT scanning were evaluated retrospectively. The inclusion criteria were as follows: (1) patients with histologically proven PTC. (2) patients with PTC treated with total or near-total thyroidectomy and postoperative ^131^I ablation. (3) postablation negative ^131^I-WBS defined by the absence of non-physiological ^131^I uptake outside the thyroid bed or abnormal ^131^I uptake confirmed for physiological uptake or contamination by ^131^I single photon emission computed tomography/computed tomography (^131^I-SPECT/CT) outside the thyroid bed. (4) negative Tg defined as Tg < 0.2 ng/mL (TSH suppression) or Tg < 1 ng/mL (after stimulation) 6 months after the first remnant ablation. (5) progressively increased TgAb level including TgAb which persistently rose or TgAb which kept stable/decreased for some time but subsequently rose after remnant ablation. (6) ^18^F-FDG PET/CT was performed more than 1 year after the first remnant ablation. The exclusion criteria were as follows: (1) Tg ≥ 0.2 ng/mL (TSH suppression) or ≥1 ng/mL (after stimulation) in the follow-up. (2) persistently high TgAb but had no rising trend (3) a temporarily increased TgAb at 6–12 months after ablation therapy.

### ^131^I empiric treatment

After surgery, each patient received an ablative dose of ^131^I and was put on a low iodine diet for 3–4 weeks before ^131^I therapy (TSH reached 30 mIU/L). Subsequently, the patients were subjected to oral administration of ^131^I after the following conventional measurements, including FT3, FT4, TSH, Tg, TgAb, neck ultrasonography (US), and CT scans. The dose of oral standard administration of 3.7GBq (100 mCi) of ^131^I was used to ablate the thyroid remnants. ^131^I-WBS and/or ^131^I-SPECT/CT fusion imaging was performed 3–5 days after ^131^I oral administration. ^131^I-WBS was performed in both anterior and posterior projections using a dual-head SPECT with High-energy collimators and a 364-keV photo peak. ^131^I-SPECT/CT images were acquired immediately after planar imaging for PTC patients who presented suspicious finding on ^131^I-WBS.

### ^18^F-FDG PET/CT Scan

Patients were instructed to fast for at least 6 hours before the injection of ^18^F-FDG. Blood glucose level was measured before injection and ^18^F-FDG was administered at glucose levels < 150 mg/dL. ^18^F-FDG PET/CT scanning was performed after an i.v. injection of 3–4MBq/Kg ^18^F-FDG, followed by a one hour uptake phase. No intravenous contrast agent was administered. ^18^F-FDG PET/CT images were performed using a dedicated GE Discovery PET/CT scanner including 64 slice CT scanners with a dedicated PET (BGO plus crystal). ^18^F-FDG images were acquired for 4 minutes at each bed position from the skull base to the superior mediastinum with patients’ arms along the chest and from the neck to the mid-thigh with patients’ arms above the head. No specific breathing instructions were given. The CT scan was obtained from the orbitomeatal line and progressed to the mid-thigh with the use of a standardized protocol involving 140 kV, 110 mA, 0.8 seconds/rotation, pitch of 1.75:1, length of scan: 1.0 to 1.6 m, 0.625 spatial resolution, and slice thickness of 3.75 mm. Attenuation correction of PET images was performed using attenuation data from CT and images reconstruction was done using a standard reconstruction algorithm with ordered subset expectation maximization (OSEM). Image fusion was performed using coordinate based fusion software and subsequently reviewed at a workstation (Xeleris) that provided multi-planar reformatted images and displayed PET, CT, and PET/CT fusion images.

### Neck US

US were performed on the day of ^131^I administration and every 3–6 months after ^131^I ablation on a high-resolution ultrasound system equipped with a high-energy 14 MHz linear probe, allowing to work in fundamental B-mode and in power Doppler mode. The thyroid bed, central and lateral neck compartments were included for neck US examination. Suspicion of lymph node metastases of PTC was based on the following criteria: hyperechoic punctuations, cystic appearance, hypervascularization, round shape node without hyperechoic hilum and a short axis greater than 7 mm^36^.

### Tg and TgAb measurement

Serum Tg and TgAb levels were measured by electrochemiluminescence immunoassay (ECLIA) methods on the Cobas analyzer (Roche Diagnostics GmbH). The analytical sensitivity was <0.1 μg/L with reference range 1.4–78 μg/L. The analytical sensitivity of TgAb is < 10 IU/mL with a reference range of 10–4000 IU/mL.

### Follow-up

Serum Tg was performed at TSH suppression or at after TSH suppression 6 months after first remnant ablation. Subsequently, FT3, FT4, TSH, Tg, TgAb at TSH suppression and neck US were measured and performed every 3–6 months in the follow-up of the period, respectively. Progressively increased TgAb levels were measured no less than three times. All ^18^F-FDG PET/CT scan were performed at TSH suppression. Contrast-enhanced CT was performed for a few patients after ^18^F-FDG PET/CT scan. The last neck US at diagnosis, contrast-enhanced CT and ^18^F-FDG PET/CT scan were performed at a maximum interval of less than 30 days. The follow-up period was 2–9 yr with a median follow-up of 5.1 yr.

### Image analysis

^18^F-FDG PET/CT images were reviewed and interpreted by 2 experienced nuclear medicine physicians (Z-L Qiu and W-J Wei). All ^18^F-FDG PET/CT were considered as negative or positive. The criterion for positive lesion was accepted as ^18^F-FDG uptake greater than that of the normal surrounding tissue or when the SUV_max_ was ≥ 2.5. The anatomical confirmation with a lesion was detected with matched CT scan. The criterion for negative lesion was that there was no ^18^F-FDG uptake and no corresponding identifiable lesion on matched CT scans.

### Evaluation of ^18^F-FDG PET/CT findings

^18^F-FDG PET/CT results were correlated with surgical and histopathological findings, ^131^I-WBS combined with ^131^I-SPECT/CT after ^131^I treatment once again, other imaging modalities including neck US, chest CT, ^99m^Tc-MDP bone scan and follow-up. A true-positive finding was confirmed when a lesion was detected as positive by ^18^F-FDG PET/CT and the patient was found to have recurrent disease by surgical pathology. A false-positive finding was confirmed when a lesion was excluded by surgical pathology in the patients with positive lesions on ^18^F-FDG PET/CT. A false-negative finding was confirmed when a lesion couldn’t be detected on ^18^F-FDG PET/CT, but it could be found to be recurrent disease by surgical pathology or by ^131^I-WBS combined with ^131^I-SPECT/CT after ^131^I treatment once again. A true-negative finding were confirmed when a lesion was detected as negative by ^18^F-FDG PET/CT and the patient was found to have benign disease by surgical pathology or it could be wasn’t found to have recurrent disease on other imaging modalities including neck US, chest CT and ^131^I-WBS and ^99m^Tc-MDP bone scan in the follow-up period.

### Statistical analysis

Statistical analyses were performed with the SPSS v.17.0 statistical package (SPSS, Inc., Chicago, IL, USA). Descriptive statistics were represented as frequency and percentage. Categorical variables were compared by Pearson Chi-square. The categorical variables for positive ^18^F-FDG PET/CT were analyzed by univariate logistic regression. The sensitivity, specificity, positive and negative predictive values, and accuracy of ^18^F-FDG PET/CT for the detection of recurrent thyroid cancer were calculated. Together with their 95% confidence intervals (CIs), the odds ratios (OR) for ^18^F-FDG PET/CT findings were calculated by univariate logistic regression. A P value of <0.05 was considered to be statistically significant and all reported P values are two-side.
